# Complete genome sequence of *Desulfurispirillum indicum* strain S5^T^

**DOI:** 10.4056/sigs.2425302

**Published:** 2011-12-22

**Authors:** Elisabetta Bini, Ines Rauschenbach, Priya Narasingarao, Valentin Starovoytov, Lauren Hauser, Cynthia D. Jeffries, Miriam Land, David Bruce, Chris Detter, Lynne Goodwin, Shunsheng Han, Brittany Held, Roxanne Tapia, Alex Copeland, Natalia Ivanova, Natalia Mikhailova, Matt Nolan, Amrita Pati, Len Pennacchio, Sam Pitluck, Tanja Woyke, Max Häggblom

**Affiliations:** 1 Rutgers, The State University of New Jersey, Department of Biochemistry and Microbiology, School of Environmental and Biological Sciences, New Brunswick, New Jersey, USA; 2 Rutgers, The State University of New Jersey, Department of Cell Biology and Neuroscience, Piscataway, NJ, USA; 3 Oak Ridge National Laboratory, Oak Ridge, Tennessee, USA; 4 Los Alamos National Laboratory, Los Alamos, New Mexico, USA; 5 Joint Genome Institute, Walnut Creek, USA; 6 Current address: Scripps Institution of Oceanography, San Diego, CA, USA

**Keywords:** *Desulfurispirillum indicum* S5, *Chrysiogenetes*, arsenate, selenate, anaerobe, free-living

## Abstract

*Desulfurispirillum indicum* strain S5^T^ is a strictly anaerobic bacterium isolated from river sediment in Chennai, India. *D. indicum* belongs to the deep branching phylum of *Chrysiogenetes*, which currently only includes three other cultured species. Strain S5^T^ is the type strain of the species and it is capable of growth using selenate, selenite, arsenate, nitrate or nitrite as terminal electron acceptors. The 2,928,377 bp genome encodes 2,619 proteins and 49 RNA genes, and the information gained from its sequence will be relevant to the elucidation of microbially-mediated transformations of arsenic and selenium, in addition to deepening our knowledge of the underrepresented phylum of *Chrysiogenetes*.

## Introduction

*Desulfurispirillum indicum* type strain S5^T^ (=DSM 22839^T^ =ATCC BAA-1389^T^) was isolated from an estuarine sediment for its ability to grow on selenate [1]. *D. indicum* belongs to the *Chrysiogenetes*, a deeply branching phylum that includes three other cultured species: *Chrysiogenes arsenatis* [2], *Desulfurispirillum alkaliphilum* [3], and *Desulfurispira natronophila* [4]. The four microorganisms are all strict anaerobes and are capable of using a variety of terminal electron acceptors and a few short-chain fatty acids as electron donors and sources of carbon. Specifically, *D. alkaliphilum* can respire sulfur, fumarate, nitrate, nitrite and chromate, while *C. arsenatis* can grow using arsenate, nitrate and nitrite. *Desulfurispira natronophila* can grow under moderate haloalkaline conditions, respiring sulfur or arsenate. Thus, *D. indicum* is the only characterized *Chrysiogenetes* that is capable of dissimilatory reduction of both arsenate and selenate, in addition to nitrate and nitrite respiration. This feature makes it an ideal system to identify and elucidate the pathways for selenate and arsenate oxyanions respiration and their regulation. Here we summarize the features of *D. indicum* and present a description of its sequenced genome, which is the first sequenced genome of a member of the phylum *Chrysiogenetes*.

## Organism information

**Figure 1:**
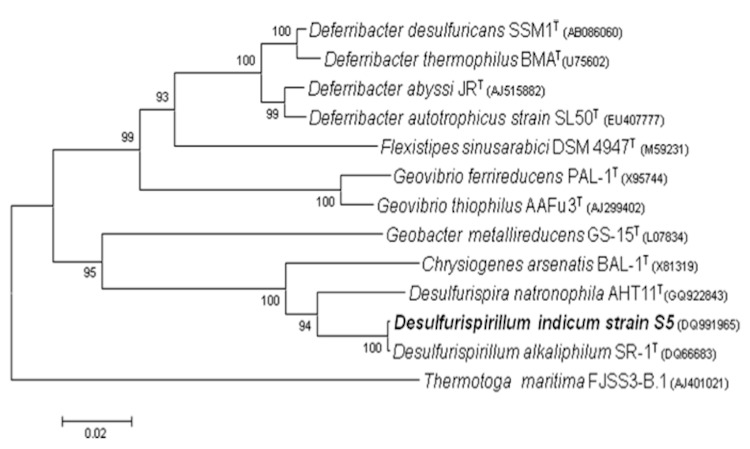
Phylogenetic tree highlighting the position of *Desulfurispirillum indicum* strain S5 relative to other type strains within the *Chrysiogenetes* and *Deferribacteres* phyla. The strains and their corresponding GenBank accession numbers for 16S rRNA genes are as indicated (type strain=^T^). The tree, based on 1,251 positions, was built with Mega 4 [14] using the Neighbor-Joining method and 1,000 bootstrap replications. *T. maritima* was used as an outgroup.

**Figure 2:**
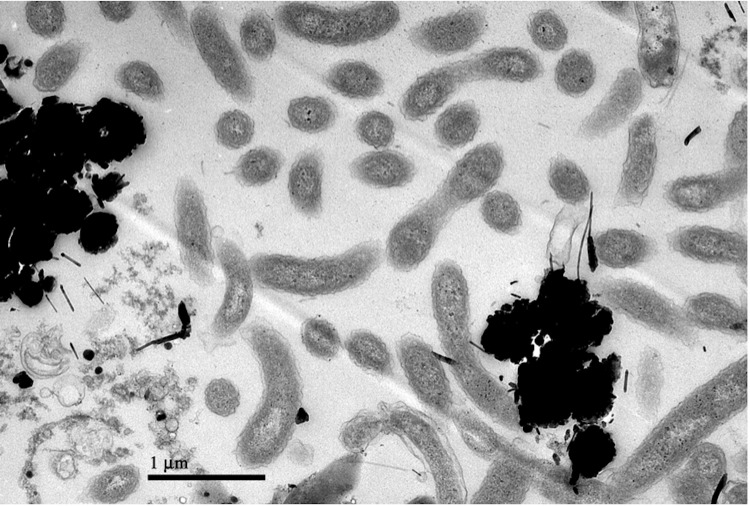
Transmission electron micrograph of *D. indicum* S5^T^.

*D. indicum* forms a deeply branching clade related to *Chrysiogenes arsenatis*, an arsenate respiring bacterium that cannot use selenate as electron acceptor, and *Desulfurispira natronophila* that only uses sulfur or arsenate as terminal electron acceptor (Table 1). Interestingly, its closest relative *D. alkaliphilum*, with a 16S rRNA gene identity of 99.8%, is not capable of either arsenate or selenate respiration. The phylogenetic position of *D. indicum* relative to its closest relatives is shown in Figure 1. This Gram-negative bacterium is spiral-shaped and accumulates electron-dense granules when grown in the presence of selenium (Figure 2).

**Table 1:** Classification and general features of *Desulfurispirillum indicum* strain S5

**MIGS ID**	**Property**	**Term**	**Evidence code**^a^
	Current classification	Domain *Bacteria*	TAS [5]
		Phylum *Chrysiogenetes*	TAS [6,7]
		Class *Chrysiogenetes*	TAS [6,8]
		Order *Chrysiogenales*	TAS [6,9]
		Family *Chrysiogenaceae*	TAS [6,10]
		Genus *Desulfurispirillum*	TAS [3,11]
		Species *Desulfurispirillum indicum* Type strain S5	TAS [12]
	Gram stain	Negative	TAS [12]
	Cell shape	Spiral (2-7 μm long, 0.10-0.15 μm in diameter)	TAS [12]
	Motility	Motile	TAS [12]
	Sporulation	Non-sporulating	TAS [12]
	Temperature range	25-37 °C	TAS [12]
	Optimum temperature	28 °C	TAS [12]
	Carbon source	Pyruvate, lactate, acetate	TAS [12]
	Energy source	Pyruvate, lactate, acetate	TAS [12]
	Terminal electron acceptor	Selenate, selenite, arsenate, nitrate, nitrite	TAS [12]
MIGS-6	Habitat	Estuarine sediment	TAS [12]
MIGS-6.3	Salinity	Tolerates NaCl concentrations up to 0.75 M	TAS [12]
MIGS-22	Oxygen	Obligate anaerobe	TAS [12]
MIGS-15	Biotic relationship	Free living	TAS [12]
MIGS-14	Pathogenicity	Not reported	NAS
MIGS-4	Geographic location	Buckingham Canal, Chepauk, Chennai, India	TAS [12]

a) Evidence codes - IDA: Inferred from Direct Assay; TAS: Traceable Author Statement (i.e., a direct report exists in the literature); NAS: Non-traceable Author Statement (i.e., not directly observed for the living, isolated sample, but based on a generally accepted property for the species, or anecdotal evidence). These evidence codes are from the Gene Ontology project [13].

## Genome sequencing information

### Genome project history

The genome of *D. indicum* strain S5 was selected for sequencing in 2007 by the DOE Joint Genome Institute as a part of the DOE JGI Community Sequencing Program. The Quality Draft (QD) assembly and annotation were completed on July 3, 2009, and presented for public access on December 31, 2009 in the ORNL database. The final complete genome was made available on September 14, 2010. Table 2 presents the project information and its association with MIGS version 2.0 compliance [15].

**Table 2:** Project information

**MIGS ID**	**Property**	**Term**
MIGS-31	Finishing quality	Finished
MIGS-28	Libraries used	454 Titanium standard, 454 Paired End, Illumina (Solexa)
MIGS-29	Sequencing platforms	454, Illumina
MIGS-31.2	Fold coverage	198× (454 data), 222× (Illumina)
MIGS-30	Assemblers	Newbler, Velvet
MIGS-32	Gene calling method	Prodigal, GenePRIMP
	Genome Database release	Jan 4, 2010 (draft)
	Genbank ID	CP002432, NC_014836
	Genbank Date of Release	Jan 6, 2011 (draft)
	GOLD ID	Gi02042
	Project relevance	Bioremediation, Biotechnological, Environmental, Biogeochemical cycling of As and Se

### Growth conditions and DNA isolation

*D. indicum* was grown in mineral salt medium at 28°C with 20 mM pyruvate as carbon source and 10 mM nitrate as electron acceptor, as previously described [12,16]. Genomic DNA was isolated from an 80-ml culture using a phenol-chloroform extraction protocol [17].

### Genome sequencing and assembly

The draft genome of *Desulfurispirillum indicum* was generated at the DOE Joint Genome Institute (JGI) using a combination of Illumina [18] and 454 technologies [19]. For this genome, we constructed and sequenced an Illumina GAii shotgun library which generated 16,867,720 reads totaling 607 Mbp, a 454 Titanium standard library which generated 234,340 reads and paired end 454 library with average insert sizes of 6, 18 and 23 Kbp which generated 475,179 reads totaling 291 Mbp of 454 data. All general aspects of library construction and sequencing performed at the JGI can be found at the JGI website [20]. The initial draft assembly contained 117 contigs in 1 scaffold. The 454 Titanium standard data and the 454 paired end data were assembled together with Newbler, version 2.3. The Newbler consensus sequences were computationally shredded into 2 Kbp overlapping fake reads (shreds). Illumina sequencing data was assembled with Velvet, version 0.7.63 [21], and the consensus sequences were computationally shredded into 1.5 Kbp overlapping fake reads (shreds). We integrated the 454 Newbler consensus shreds, the Illumina Velvet consensus shreds and the read pairs in the 454 paired end library using parallel phrap, version SPS - 4.24 (High Performance Software, LLC). The software Consed [22-24] was used in the following finishing process: Illumina data was used to correct potential base errors and increase consensus quality using the software Polisher developed at JGI (Alla Lapidus, unpublished). Possible mis-assemblies were corrected using gapResolution (Cliff Han, unpublished), Dupfinisher [25], or sequencing cloned bridging PCR fragments with subcloning. Gaps between contigs were closed by editing in Consed, by PCR and by Bubble PCR (J-F Cheng, unpublished) primer walks. A total of 764 additional reactions were necessary to close gaps and to raise the quality of the finished sequence. The total size of the genome is 2,928,377 bp and the final assembly is based on 220 Mbp of 454 draft data which provides an average 108 × coverage of the genome and 607 Mbp of Illumina draft data which provides an average 222 × coverage of the genome.

### Genome annotation

Genes were identified using Prodigal [26] as part of the Oak Ridge National Laboratory genome annotation pipeline, followed by a round of manual curation using the JGI GenePRIMP pipeline [27]. The predicted CDSs were translated and used to search the National Center for Biotechnology Information (NCBI) nonredundant database, UniProt, TIGRFam, Pfam, PRIAM, KEGG, COG, and InterPro databases. These data sources were combined to assert a product description for each predicted protein. Non-coding genes and miscellaneous features were predicted using tRNAscan-SE [28], RNAMMer [29], Rfam [30], TMHMM [31], and signalP [32].

## Genome properties

The genome includes a single circular chromosome of 2,928,377 bp (56.1% GC content). In total, 2,668 genes were predicted, 2,619 of which are protein-coding genes. Of these, 2,137 protein coding genes were assigned to a putative function while those remaining were annotated as hypothetical proteins. 91 protein coding genes belong to 25 paralogous families in this genome corresponding to a gene content redundancy of 3.4%. The properties and the statistics of the genome are summarized in Table 3 and Table 4.

**Table 3:** Nucleotide content and gene count levels of the genome

**Attribute**	**Value**	**% of total^a^**
Genome size (bp)	2,928,377	100
DNA coding region (bp)	2,598,759	88.74
G+C content (bp)	1,643,075	56.11
Total genes^b^	2,668	100
RNA genes	49	1.84
Protein-coding genes	2,619	98.16
Genes in paralog clusters	91	3.41
Genes assigned to COGs	2,137	80.10
Genes with signal peptides	851	31.90
Genes with transmembrane helices	673	25.22
Paralogous groups	25	N/A

a) The total is based on either the size of the genome in base pairs or the total number of protein-coding genes in the annotated genome.

b) Also includes 49 RNA genes. It does not include 48 pseudogenes.

**Table 4:** Number of genes associated with the general COG functional categories

**Code**	**Value**	**% age**^a^	**Description**
J	149	6.24	Translation
K	116	4.86	Transcription
L	143	5.99	Replication, recombination and repair
B	1	0.04	Chromatin structure and dynamics
D	34	1.42	Cell cycle control, mitosis and meiosis
V	36	1.51	Defense mechanisms
T	259	10.85	Signal transduction mechanisms
M	148	6.20	Cell wall/membrane biogenesis
N	120	5.03	Cell motility
Z	1	0.04	Cytoskeleton
U	85	3.56	Intracellular trafficking and secretion
O	98	4.10	Posttranslational modification, protein turnover, chaperones
C	167	6.99	Energy production and conversion
G	73	3.06	Carbohydrate transport and metabolism
E	148	6.20	Amino acid transport and metabolism
F	59	2.47	Nucleotide transport and metabolism
H	128	5.36	Coenzyme transport and metabolism
I	54	2.26	Lipid transport and metabolism
P	143	5.99	Inorganic ion transport and metabolism
Q	24	1.01	Secondary metabolites biosynthesis, transport and catabolism
R	228	9.55	General function prediction only
S	174	7.29	Function unknown
-	531	19.90	Not in COGs

a) The total is based on the total number of protein coding genes in the annotated genome.

## Discussion

**Figure 3:**
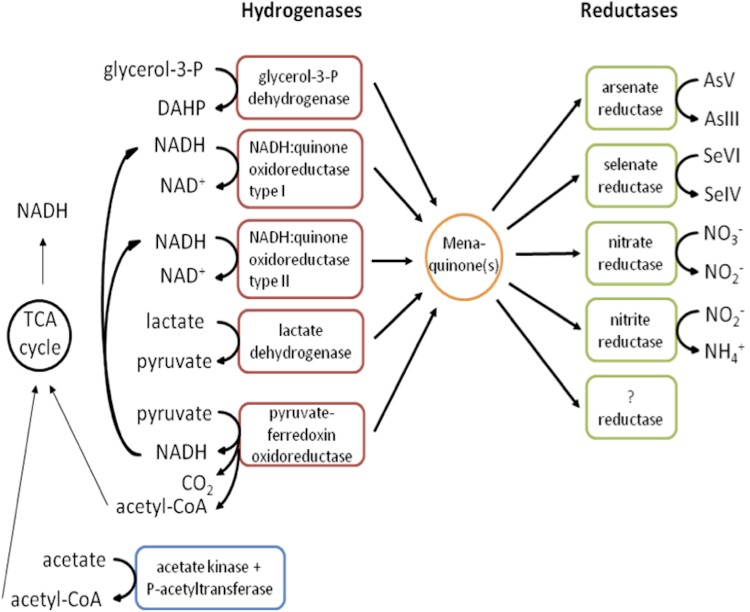
Diagram of the anaerobic pathways of respiration in *D. indicum* strain S5, based on genomic and physiology data. Question mark indicates the presence of sequences encoding terminal reductases whose substrate is unknown.

*D. indicum* strain S5 can use nitrate, nitrite, arsenate or selenate as the terminal electron acceptors for growth, while using the electron donors acetate, lactate or pyruvate [12,33]. The inspection of the strain S5 genome has confirmed the physiological data, and furthermore has enabled the discovery of sequences encoding other DMSO-like terminal reductases, as well as enzymes for the oxidation of additional electron donors ( [33] and Fig. 3). The discovery of such sequences suggests that the respiratory capabilities of strain S5 are broader than expected, and allows us to formulate hypotheses on further substrates and TEAs to be tested. In particular, we are interested in the dissimilatory reduction of selenium and arsenic oxyanions. Although the reduction of selenium is an important mode of respiration, the genes responsible for this process remain largely uncharacterized and virtually nothing is known about their regulation, or their interactions with other respiratory pathways.

Besides *Desulfurispirillum indicum*, the genomes of only four bacterial species capable of using selenate reduction for growth are currently available: *Aeromonas hydrophila* [34], *Desulfitobacterium hafniense* [35], *Sulfurospirillum barnesii* [36] and *Thauera selenatis* [37,38]) [12]. The genome of the selenite respirer *Bacillus selenitireducens* [39] has also been sequenced. Comparisons of the DMSO-like sequences from these genomes will help to generate testable hypotheses about functions and substrates of the various terminal reductases.
